# A dataset mapping the geographical distribution of past and present collective farmers’ stores in France

**DOI:** 10.1016/j.dib.2026.112475

**Published:** 2026-01-20

**Authors:** Grégori Akermann, Manon Pradère

**Affiliations:** INRAE Montpellier, UMR Innovation

**Keywords:** Agriculture, Alternative food networks (AFN), Short food supply chain, Crowdsourcing, collective farmers’ stores

## Abstract

Collective farmers' stores (CFS) represent in France a unique model within alternative food networks, characterized by a collective governance exclusively managed by a group of farmers. These stores saw substantial growth in the country during the 2010–2020 decade. However, the post-COVID period, affected by inflation, appears to have sparked a wave of closures. To our knowledge, no systematic tracking of CFS openings and closures has been conducted in recent years in France, nor has any related data been made available for research purposes. Our objective was to identify all stores that have recently opened or closed, in order to analyze the expansion dynamics of this unique food retail model. By cross-referencing these data with other spatial factors — such as demographic, economic, and public policy information — it becomes possible to explore research questions that shed light on the determinants influencing the establishment or disappearance of these stores across different territories. We identified collective farmers’ stores and their location throughout France using three complementary methods: analyzing local daily newspapers, gathering participatory data through an interactive map, and sharing data via established partnerships with agricultural development organizations. We aimed to associate each store with its SIRET number (French business identification number) to enrich the data with opening and closure dates, as well as relevant administrative and economic details. Our final dataset includes 543 active stores in 2024, along with 105 that have closed over the past 15 years [1].

Specifications Table

**Table:** 

Subject	Social Sciences
Specific subject area	Alternative food networks and short food supply chains; spatial analysis of collective farmers' stores in France
Type of data	Table Raw
Data collection	The collective farmers’ stores were identified using three methods: (1) analyzing local daily newspapers, (2) collaborating with CFS networks to share data, (3) gathering information through an online crowdsourcing map developed in partnership with UFC Que Choisir, a French consumer association. The dataset was then refined and updated by cross-referencing information from publicly available business databases, such as SIRENE (the French System for the Identification of Companies and their Establishments) and BODACC (the Official Bulletin of Civil and Commercial Notices), as well as by consulting the official websites of the CFS.
Data source location	Country: France Institution: National Research Institute for Agriculture, Food and Environment (INRAE)
Data accessibility	Repository name: Entrepôt Recherche Data Gouv - UMR Innovation DOI: 10.57745/YX1JGK Direct URL to data: https://entrepot.recherche.data.gouv.fr/dataset.xhtml?persistentId=doi:10.57745/YX1JGK
Related research article	None

## 1 Value of the Data

• To our knowledge, there are no publicly accessible and regularly updated datasets in France on short food supply chain actors, particularly on collective farmers' stores, even though these supply chains represent an important lever for transitioning toward more sustainable food systems. Existing national databases primarily function as consumer-oriented directories. They aim for neither the exhaustiveness nor the detailed characterization necessary for statistical analysis, thereby hindering rigorous quantitative research.
• The multiple variables in this dataset enable the analysis of spatial and temporal distribution of collective farmers' stores, as well as the exploration of the diversity of legal structures chosen by these organizations. For instance, the opening and closure dates allow for the modeling of survival rates and failure risks as a function of lifespan, and the integration of territorial characteristics or legal statuses into the model.
• This dataset is valuable for researchers in sociology, geography, economics, political science, and agronomy to explore: the diversification of sales models; their role in transforming foodscapes; the factors driving the establishment of CFS; the effects of local food policies on the development of these stores; and the relation between agronomic characteristics of territories and the dynamism of short food supply chains. This includes modeling the territorial determinants of CFS establishment:
  ○ For instance, to what extent does the presence of an ecosystem of farms engaged in short food supply chains and organic production constitute a prerequisite for CFS establishment, versus the simple density of agricultural activity at the inter-municipal (EPCI) level?
  ○ How do the socio-demographic characteristics of local consumers (e.g., education level, income brackets) and population density (rural/urban) interact to create a demand that determines the successful establishment of CFS?
  ○ How does the legal form (e.g., Limited Liability Company, Simplified Joint-Stock Company, Association) influence the long-term longevity and resilience of the collective model?
  ○ What is the influence of public policies (such as the presence of Territorial Food Projects) on creation dynamics or the limitation of closures in the post-Covid period?
• Local authorities and agricultural development stakeholders can also mobilize this dataset to locate CFS within their territories, thereby facilitating connections between agricultural and food actors and ensuring the development of initiatives tailored to specific territorial contexts. Specifically, the data enables them to identify underserved areas suitable for the establishment of new CFS based on proximity to existing stores or population density, to assess the maturity and density of the local short food supply chain network, and to monitor the impact of development, support, and networking programs.

## 2 Background

Alternative food networks constitute a lever for the transition of food systems toward greater sustainability [2,3]. They encompass a wide range of short food supply chains (with no more than one commercial intermediary), such as Community Supported Agriculture (CSA), farmers' markets, digital direct-to-consumer platforms, and collective farmers' stores. More broadly, short food supply chains constitute social innovations contributing to the re-territorialization of agricultural and food systems [4]. Since the 2000s, multiple initiatives seeking to reconnect agriculture, food, and territories have developed in France, driven by a diversity of actors and supported by public policies such as the Barnier Plan in 2009 or the Territorial Food Projects introduced in 2014 [5].

Among these initiatives, collective farmers' stores, also known as “collective points of sale”, occupy a unique place, emblematic of the collective reclaiming of marketing activities by groups of producers [6]. In France, collective farmers' stores, referred to as “*magasins de producteurs*”, are defined in Law No. 2014-344 of March 17, 2014 [7], on consumer affairs as physical retail spaces managed by a collective of farmers who sell their own products directly “at least 70 % of total sales. These stores are characterized by collective governance and capital control by the farmers themselves. Although collective farmers’ stores serve as a pathway for renewing collective action and improving the social and economic performance of local food systems [8], they have remained under-researched compared to other forms of short food supply chains such as CSAs or farmers' markets, particularly because this model is relatively uncommon outside of France. Recent studies highlight that they constitute a singular form of collective action, at the crossroads of economic, social, and territorial logics [6]. These stores offer farmers the possibility to pool costs, skills, and time associated with direct selling, while reinforcing their commercial autonomy and visibility within the territory.

However, as in many studies on short food supply chains, the spatial and temporal dynamics of store establishment remain very poorly documented. Although a few recent studies have begun to analyze the spatial dimension of alternative short food supply chains [9], these works remain rare and limited to specific geographical contexts. National databases such as SIRENE or OpenStreetMap present limitations in terms of reliability and completeness for mapping food retail outlets [10]. Recent efforts have improved the use of the SIRENE database to inventory establishments in the food retail sector (NAF code 47) [11], but this approach is limited to businesses whose main activity is classified as food retail. It does not resolve the difficulties in identifying points of sale with more diverse legal and organizational forms, which are often registered in categories other than sector 47. For instance, 30 % of the collective farmers' stores in our database fall under activity sectors other than retail trade (NAF 47) (Fig. 4). The scientific community therefore lacks accessible, reliable, comprehensive, and regularly updated data specifically focused on these collective points of sale at the national scale.

To address this gap, INRAE established the Observatory of Territorialized Food Systems (ObSAT) [12] in 2022, in collaboration with a range of stakeholders gathered within the Mixed Technological Network 'Local Food' [13]. As part of the development of this observatory, we designed a methodology for collecting data on collective farmers' stores, combining crowdsourcing, press analysis, establishing partnerships with regional networks, and cross-referencing with public databases (SIRENE) [14].

## 3 Data Description

This dataset provides comprehensive information on French collective farmers’ stores across France. The dataset contains 648 CFS, including 543 active and 105 inactive establishments with detailed geographic, administrative, legal, and operational information. Each establishment is uniquely identified by an ID code (*id_CFS*) and includes temporal data regarding opening and closure years, current operational status, precise location information, and legal business classification details.

The dataset is structured as a single table with 22 variables (columns) for each of the 648 establishments. These include identification, temporal, geographical, administrative, legal, and business variables. The complete list and detailed descriptions of all variables are provided in Appendix A (Table A1) and are also available in the data dictionary file of the deposited dataset [1].

### 3.1 Data Completeness and Quality

The dataset shows varying degrees of completeness and data quality across variables.

Core identifiers, such as the year of opening and precise address details, have a near-complete coverage rate of 99.8 %. The ***year_opening*** variable is derived from four different sources, which follow two distinct definition:

• years of opening collected from local daily newspapers, collective farmers’ stores websites, and direct contact with stores (via email or phone) correspond to the year the establishment first opened to the public.
• years of opening sourced from the SIRENE database refers to the year the establishment was legally created.

Our primary objective with this variable was to capture the first definition.

For 57.8 % of businesses where we collected both, the legal creation year matched the store’s actual opening year, which led us to consider SIRET data as a reliable equivalent. For the remaining cases, there was an average absolute difference of 4.5 years. Several scenarii can explain this discrepancy:

• Some establishments operate as associations, and registered as such when the project of creating a CFS emerged, sometimes years before the official opening of the store.
• The legal establishment linked to the store may change while remaining under the same legal entity, likely for juridical or administrative reasons. When the only change was the SIRET number (with the store’s name, type of collective governance and public presence remaining the same), we considered it to be the same store and did not add a new entry to our dataset. However, in such cases, the creation year recorded in SIRENE reflects the establishment’s administrative change, rather than the store’s initial public opening. This shift can, in some instances, occur several decades after the store first opened.

When aggregating data from multiple sources in order to create the *year_opening* variable, we therefore prioritized information coming from local daily newspapers, collective farmers’ stores websites and direct contact with stores. Among the 647 *year_opening* values, 37.9 % were obtained from one of these three sources.

The ***year_closure*** variable is derived from four different sources as well: local daily newspapers, CFS websites, the SIRENE database, and the BODACC database. As with *year_opening*, these sources follow two distinct definitions:

• The first two (newspapers and CFS websites) refer to the year the store was no longer accessible to consumers.
• The last two (SIRENE and BODACC) refer to the year the store was legally shut down.

However, unlike *year_opening*, the comparison between these definitions reveals much smaller discrepancies.

In this case, using multiple sources is primarily beneficial for keeping the database up to date regarding active and inactive stores. In SIRENE, the closure dates are usually recorded 2 to 3 years after the effective closure of the establishment, while the BODACC database is updated several times a week. Meanwhile, newspapers and websites give the most precise information on when customers lost access to the store, though compiling this data is more challenging.

To verify that establishments were indeed closed, and that the “inactive” status observed on SIRENE was not due to an outdated SIRET number, we conducted individual checks for each potential closure. When aggregating data from these various sources, we prioritized newspapers and websites, followed by BODACC, and lastly, SIRENE. As each store was verified individually, a significant proportion (85.7 %) of the 105 *year_closure* values are derived from newspapers and CFS websites.

The source of an ***address*** is usually the same as the source of the related collective farmers’ store’s identification. However, in cases where addresses from farmers directories or the crowdsourcing map were imprecise (e.g., a street without a number), we supplemented them with data from SIRENE. Of the addresses, 86.9 % have precision down to the street number, while only 2.0 % lack precision beyond the municipal level.

Geographic coordinates (***longitude*** and ***latitude***) are fully available, derived from the *address* variable, and exhibit the same levels of precision as indicated in the *location_precision* column. Querying the SIRENE API allowed us to collect coordinates for 69.4 % of the CFS with SIRET numbers, while the remaining coordinates were obtained by querying the adresses.data.gouv API.

93.4 % of collective farmers’ stores are associated with a ***siret_number***. Querying the SIRENE API using the names of CFS and postal codes resulted in linking 52.1 % of establishments to a SIRET number. However, 10 % of these initial associations were later revised after manually checking discrepancies between the *year_opening* across different sources. In most cases, the legal unit had been correctly identified, but not the establishment itself. More than half of the values in the *siret_number* column were obtained by consulting specialized business information platforms such as verif.fr, societes.com, and infogreffe.fr. Therefore, the information in this column should be of high quality, as it has been thoroughly verified, with discrepancies manually corrected and missing data filled in.

The legal status and business classification variables are derived from querying the SIRENE API using the *siret_number*, and therefore exhibit the same level of completeness.

The majority of establishments, representing 83.7 % of identified CFS, are recorded as active with no recorded closing year.

Incomplete data is marked as “NA” in the dataset.

### 3.2 Data Visualization and Spatial Distribution

Table 1 presents the distribution of collective farmers' stores across each French region. The Auvergne-Rhône-Alpes region stands out significantly with the highest number of active stores. As for Nouvelle-Aquitaine, it is the region that has experienced the greatest decline, with the largest number of closures over the past 15 years.

**Table 1:** Number of collective farmers' stores identified by French region.

French Regions	Nb past and present CFS	Nb closures [2010 –2024]	Nb active CFS in 2024
Auvergne-Rhône-Alpes	173	18	155
Bourgogne-Franche-Comté	29	3	26
Bretagne	39	9	30
Centre-Val de Loire	23	4	19
Corse	3	0	3
Grand Est	55	9	46
Hauts-de-France	19	1	18
Île-de-France	2	0	2
Normandie	24	4	20
Nouvelle-Aquitaine	106	22	84
Occitanie	93	15	78
Pays de la Loire	45	13	32
Provence-Alpes-Côte d'Azur	37	7	30
**Total**	**648**	**105**	**543**

**Fig. 1:**
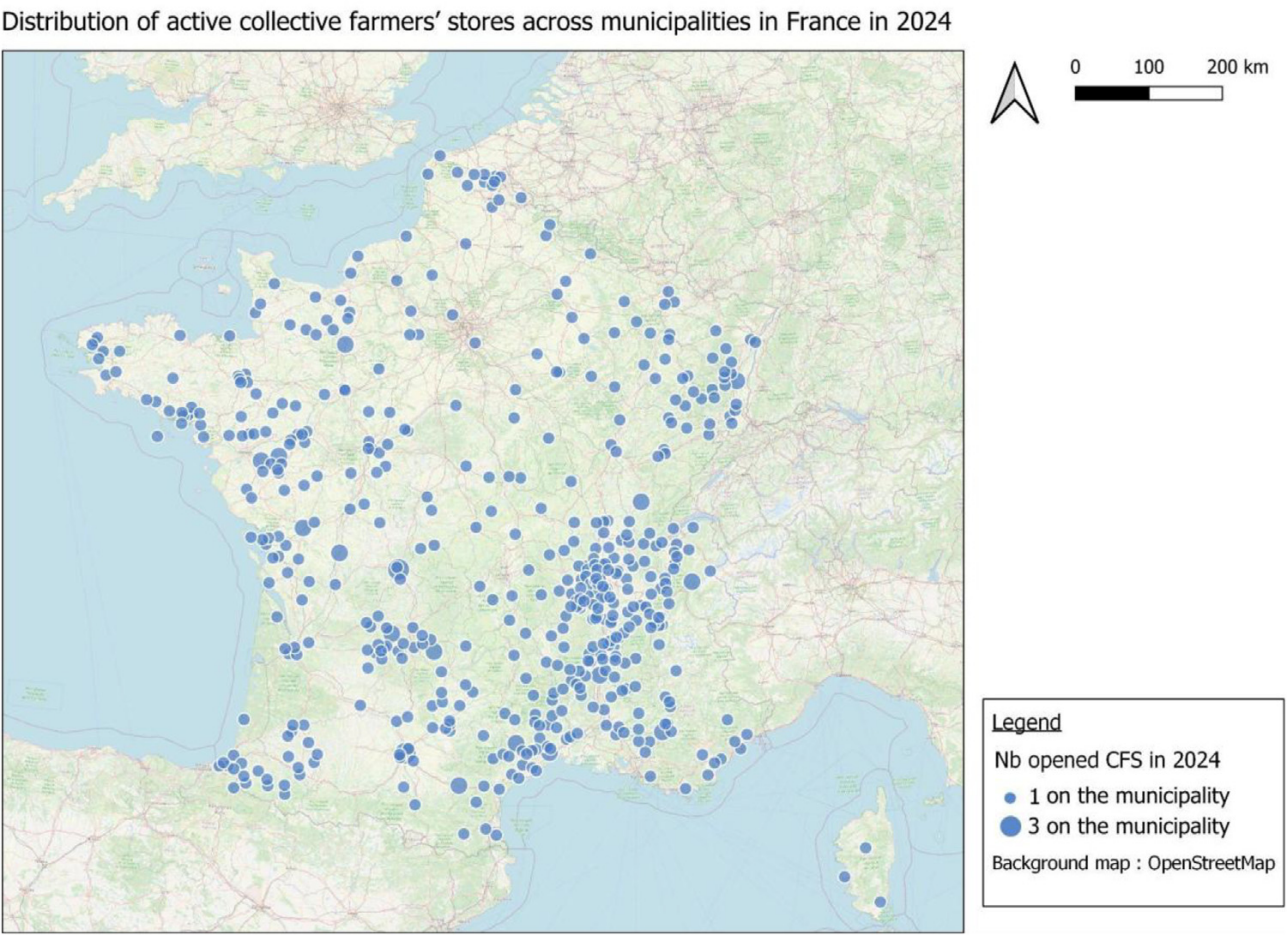
Distribution of active collective farmers’ stores across municipalities in France in 2024.

Fig. 1 illustrates the distribution and number of active collective farmers' stores per municipality in 2024. This map was created by counting the number of active CFS in each municipality and linking the data to the “Commune” shapefile from the Admin Express range, produced by the French National Institute of Geographic and Forest Information.

**Fig. 2:**
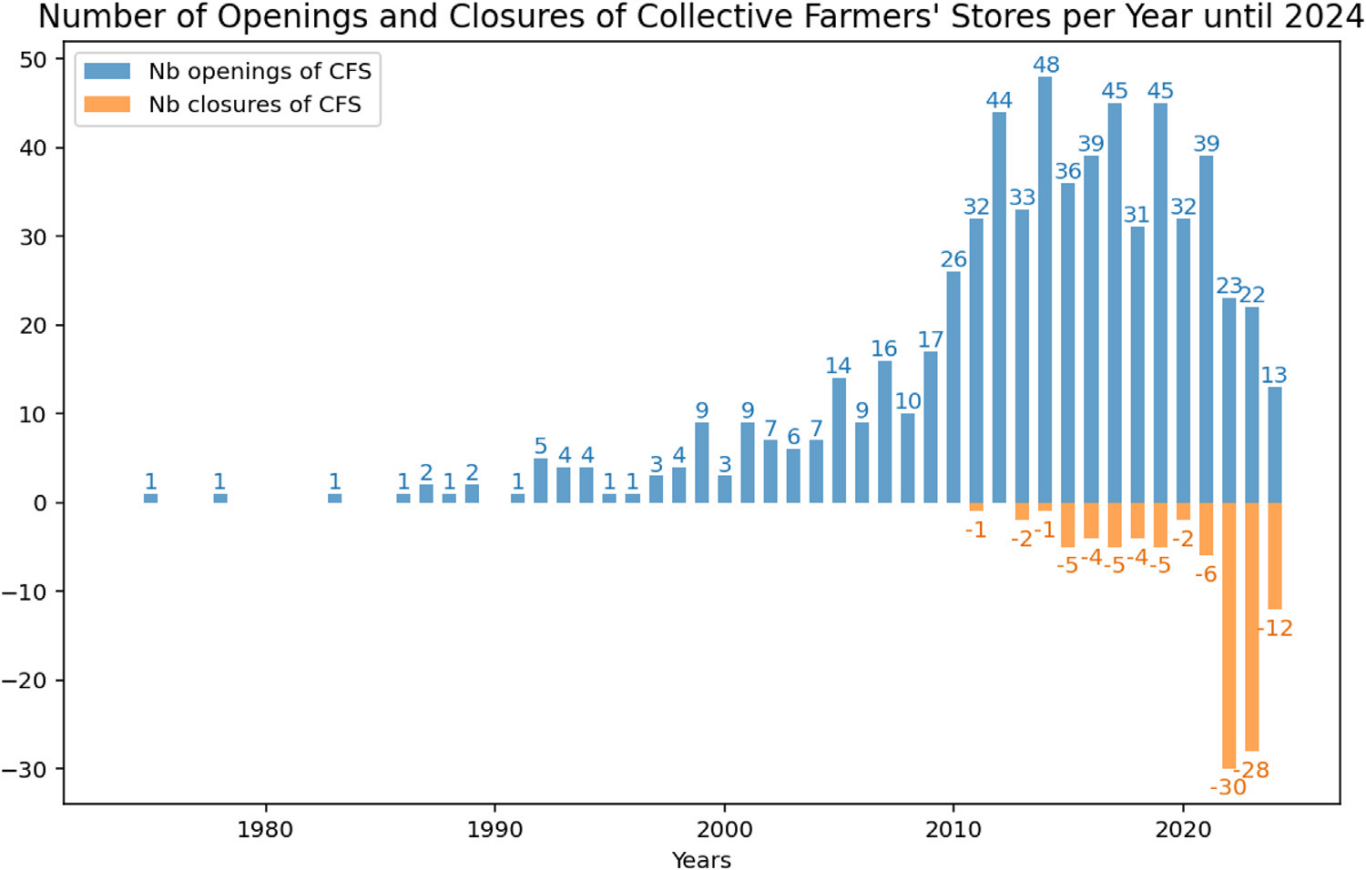
Number of openings and closures of collective farmers’ stores per year until 2024.

Fig. 2 shows the annual number of openings and closures of collective farmers' stores. The period from 2010 to 2021 experienced the highest number of store creations. The years 2022, 2023, and 2024 saw the highest number of store closures.

**Fig. 3:**
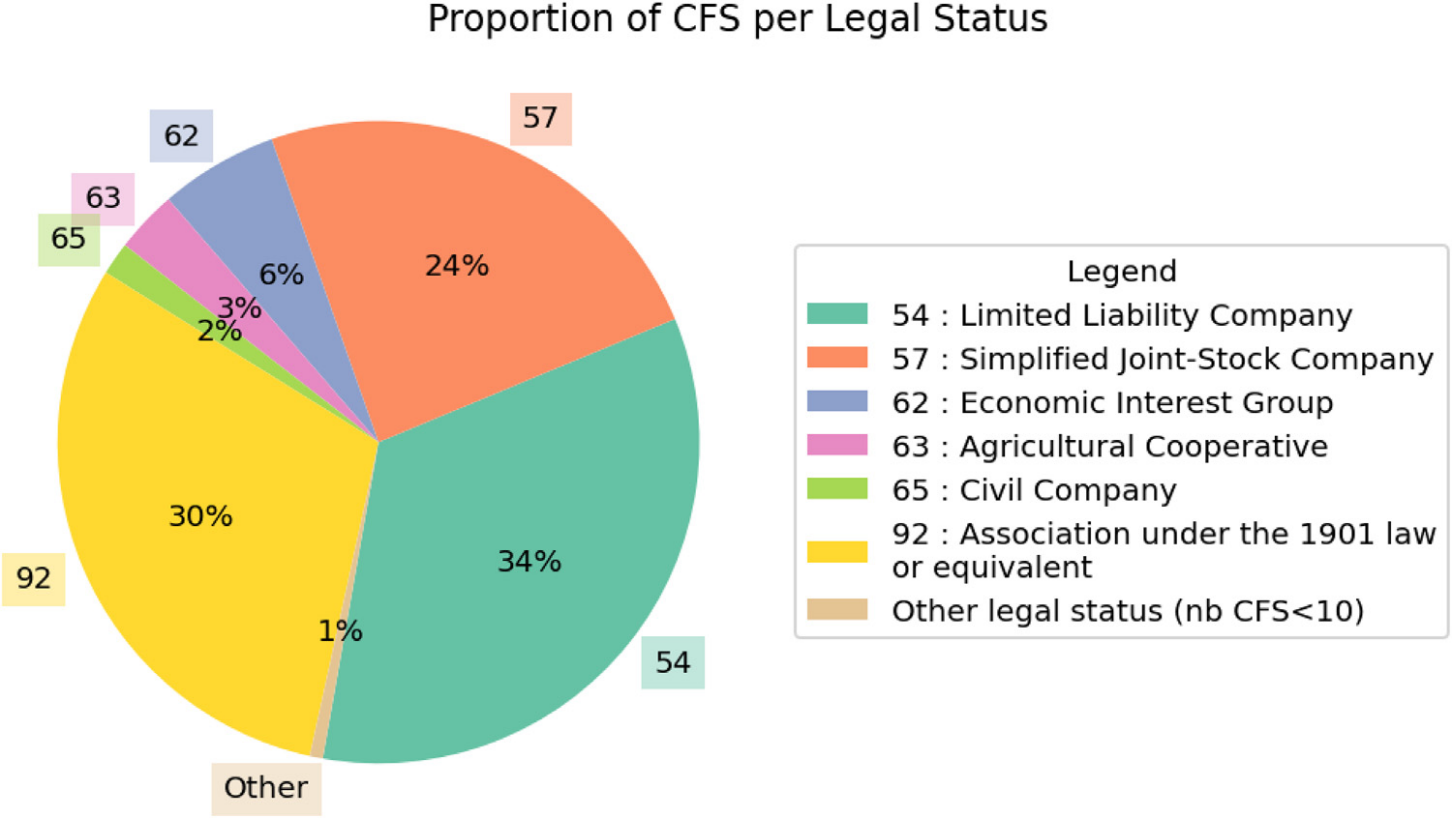
Proportion of collective farmers’ stores per legal status.

Enriching the data with variables from the SIRENE database allowed us to access each store’s legal classification variables. Fig. 3 illustrates that 88 % of CFS fall under three legal statuses: Limited Liability Company, Association under the 1901 law or equivalent, and Simplified Joint-Stock Company.

**Fig. 4:**
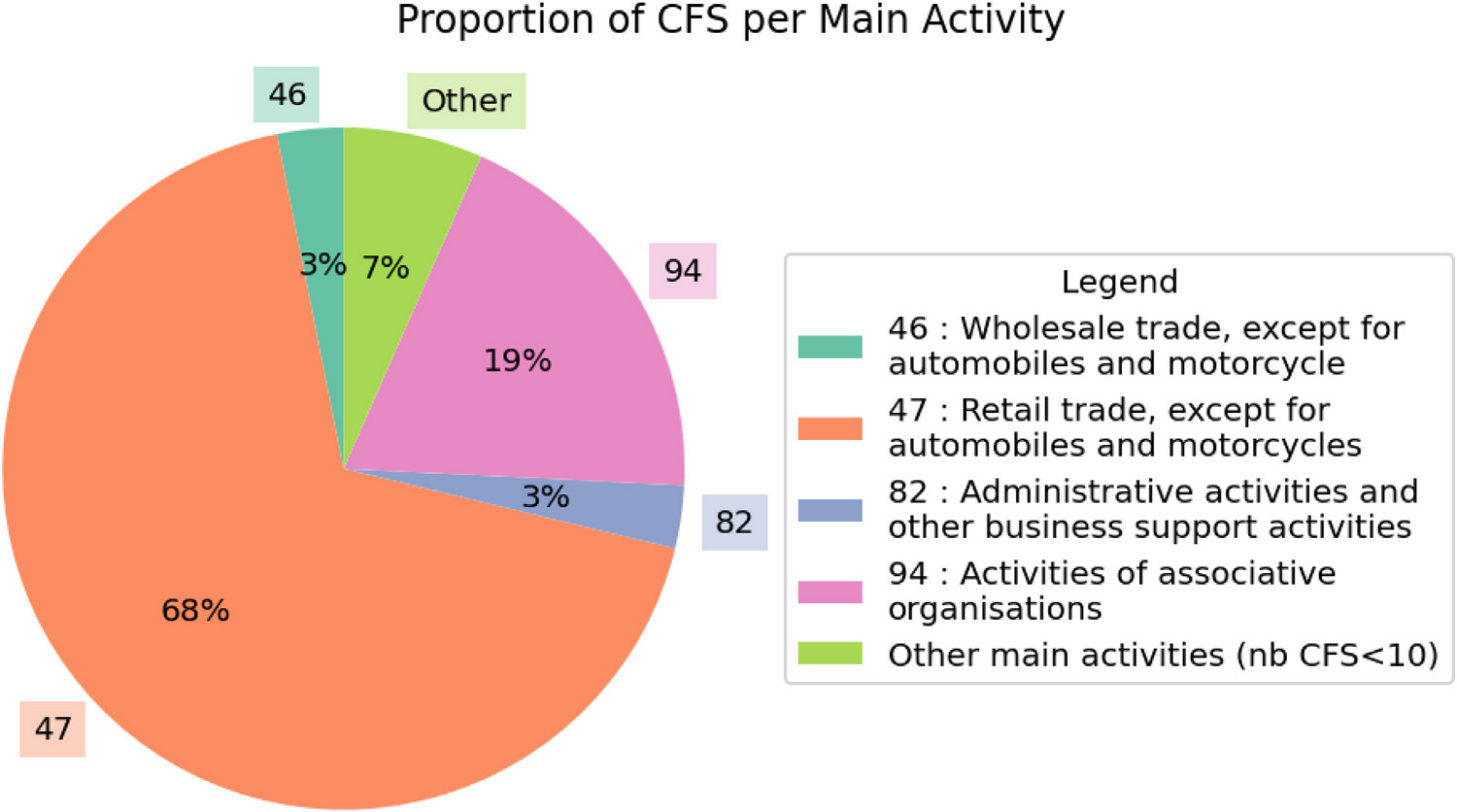
Proportion of collective farmers’ stores per main activity.

Analysis of the *naf_lib_establishment* variable reveals that only two-thirds of the CFS’s main activity is classified as retailing (Fig. 4). The remaining stores’ main activities are categorized as ‘Activities of associative organizations’, as well as under less expected sectors, such as 'Administrative activities and other business support activities' or 'Real estate activities'.

**Fig. 5:**
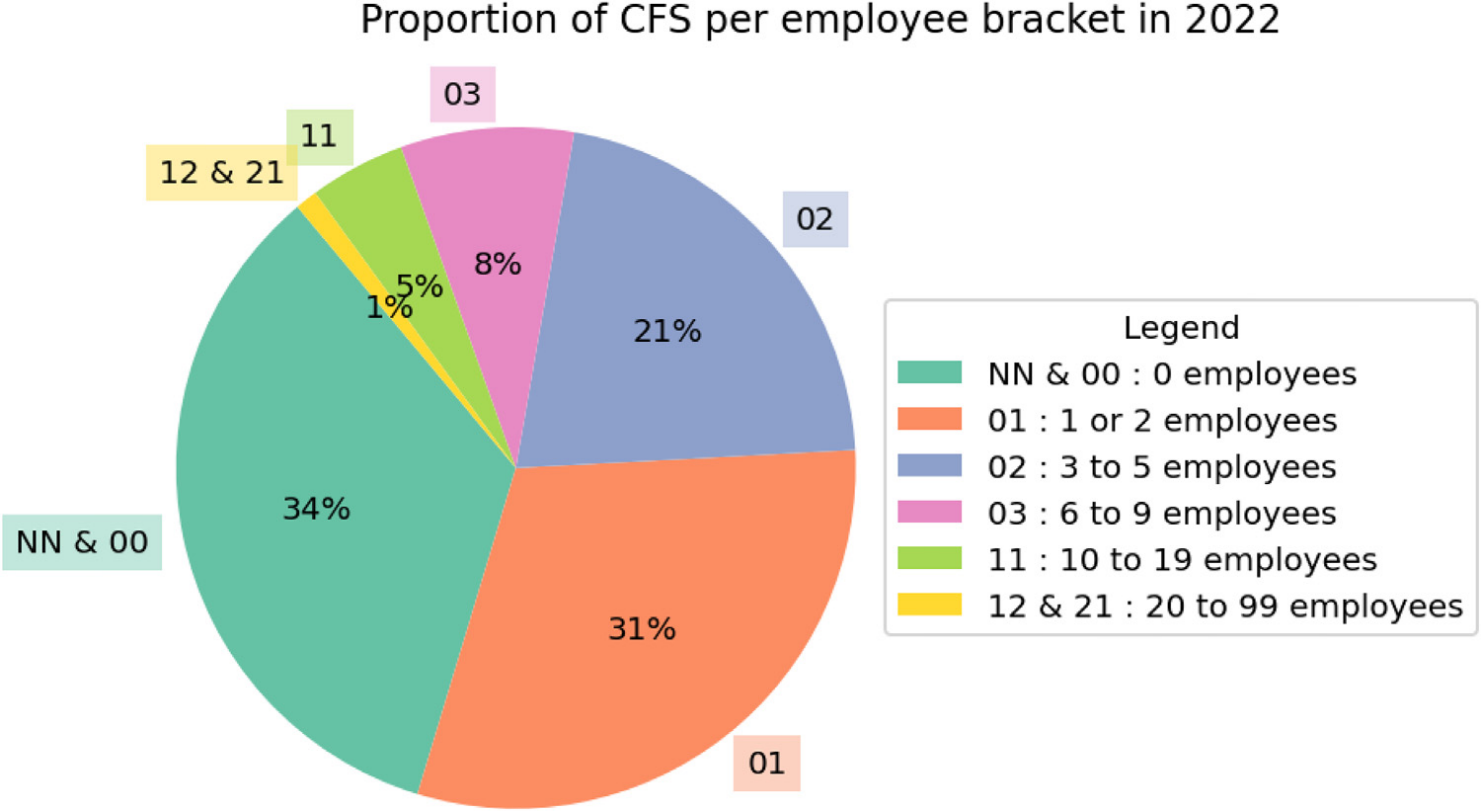
Proportion of collective farmers' stores employee bracket in 2022.

Fig. 5 shows that collective farmers' stores are mostly businesses that employ staff. However, about one-third of the active stores didn’t have any employees in 2022. In these stores, it is highly likely that the farmer-managers themselves are responsible for directly selling the products to customers.

## 4 Experimental Design, Materials and Methods

Our objective was to identify all past and present collective farmers' stores in France using data from multiple sources, and to characterize them, with a particular focus on the SIRENE database, the French System for the Identification of Businesses. In the absence of an existing database suitable for research, we developed a robust methodology consisting of five sequential steps, interspersed with data enhancement cycles.

**Fig. 6:**
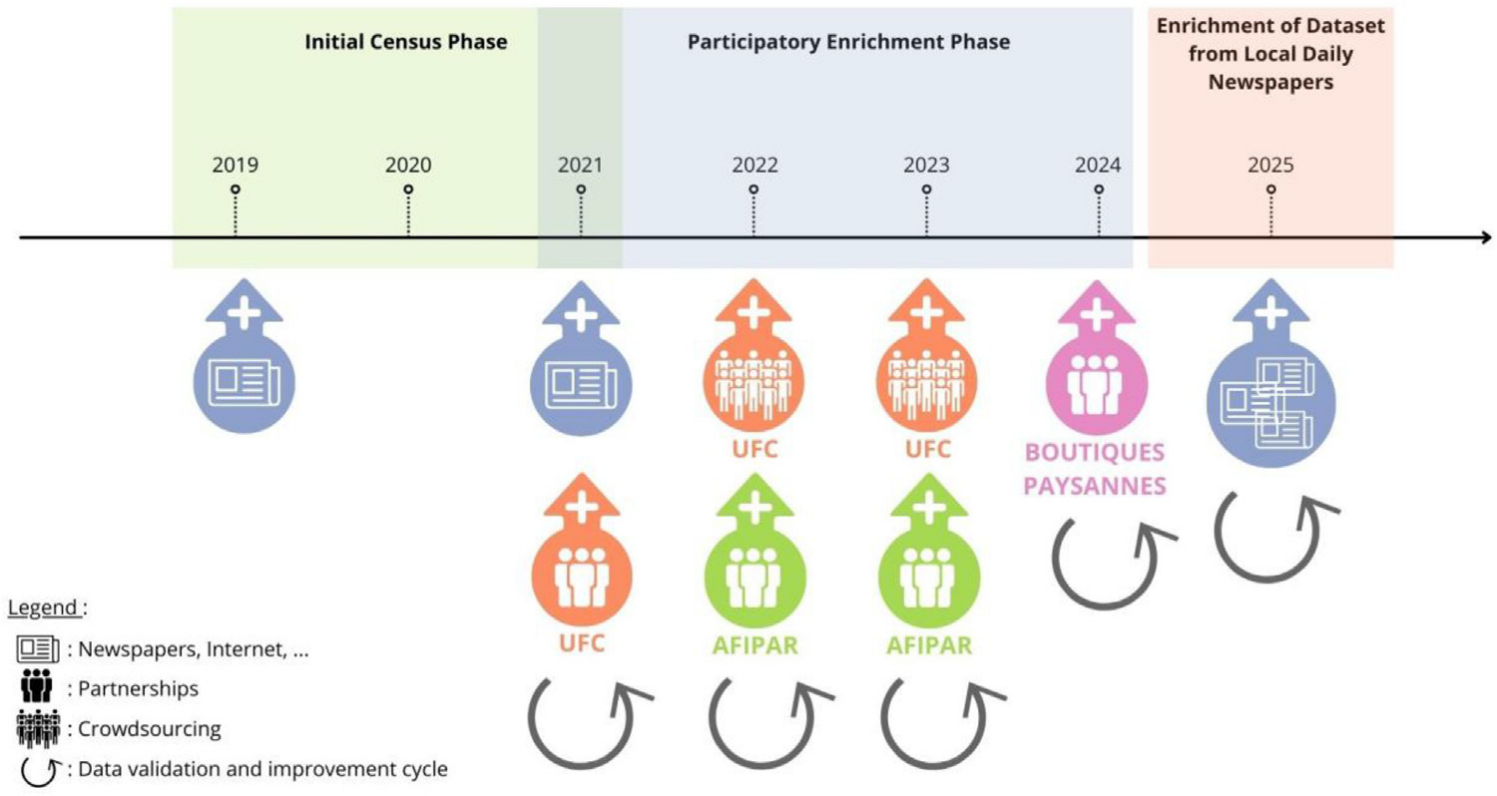
Chronology and multi-source methodology for identifying collective farmers' stores.

This timeline (Fig. 6) illustrates the sequential phases (Initial Census, Participatory Enrichment, Press Enrichment) and the active involvement of various data sources and partnerships (Internet/Newspapers, UFC Que Choisir, AFIPAR/Réseaux des Boutiques Paysannes) over the period 2019–2025.

### 4.1 Initial Census Phase (2019–2021)

**Step 1: Preliminary Identification (2019 and 2021)**

We conducted an initial identification of collective farmers' stores through several channels: Internet searches, consultation of farmers directories, and analysis of newspaper articles via the Europresse aggregator. This phase allowed us to compile a preliminary list of 409 establishments identified as CFS, along with details such as their postal addresses and website URLs.

This step was renewed in 2021 with the addition of 39 new CFS to the dataset, bringing the total to 448.

### 4.2 Participatory Enrichment Phase (2021–2024)

**Step 2: Partnership with UFC Que Choisir (2021)**

We established a collaboration with the consumer association UFC Que Choisir to develop an interactive map of short food supply chain retail outlets. In December of 2021, we provided a geolocated database of 822 stores, including our 448 collective farmers' stores. This list was submitted to UFC Que Choisir's 130 local branches for verification and enrichment. Their members proposed 617 new establishments, of which 46 were validated as collective farmers' stores following our verification process.

**Step 3: Crowdsourcing via participatory map (2022–2023)**

In February of 2022, UFC Que Choisir published a participatory map on their website listing 890 stores [15], including 432 collective farmers' stores^1^. Website users were invited to report establishments missing from the map through an online form, or suggest modifications to the data of an already listed store. We processed and moderated the data submitted via the crowdsourcing map when a sufficient number of additions and modifications were suggested, continuously enriching our dataset (Table 2).

**Table 2:** Number of new CFS identified in each update from the crowdsourcing map.

Updates	Crowd for the crowdsourcing	Year-Month	Number of new CFS identified (before deduplication on our part)
Update 0	Members of UFC Que Choisir	2021–12	46
Update 1	Website users	2022–04	19
Update 2	Website users	2022–05	35
Update 3	Website users	2022–06	8
Update 4	Website users	2023–01	22
Update 5	Website users	2023–04	15

**Step 4: Partnership with agricultural development organization (2022–2024)**

We partnered with AFIPAR and Réseaux des Boutiques Paysannes, two agricultural development organizations supporting collective farmers' stores in the Nouvelle-Aquitaine [16] and Occitanie [17] regions, respectively. This first collaboration enabled us to add 21 CFS to our database (17 in 2022 and 4 in 2023), while the second contributed 3 additional stores in 2024.

### 4.3 Enrichment of Dataset from Local Daily Newspapers (2025)

**Step 5: Building a press corpus to complete the dataset (2025)**

Due to insufficient contributions on the crowdsourcing map in 2024 for UFC Que Choisir to initiate an update of the database we share with them, we undertook an analysis of local daily newspaper articles to supplement our dataset. Utilizing the Europress aggregator, we searched for articles published between April 2023 and February 2025 that included the terms “*ouverture*” (opening) and “*magasin de producteurs*”, or “*nouveau magasin*” (new store) and “*magasin de producteurs*”. This process resulted in the compilation of a little over 200 articles and the identification of 21 new collective farmers' stores that opened in 2023 and 2024.

### 4.4 Data Validation and Improvement Cycle (periodical, from 2021 to 2025)

As part of an active observatory and at the heart of several partnerships, we made it a priority to keep our dataset up to date throughout the years. Our data enhancement cycle was performed with each major update, as well as annually in December from 2021 to 2024.

**Fig. 7:**
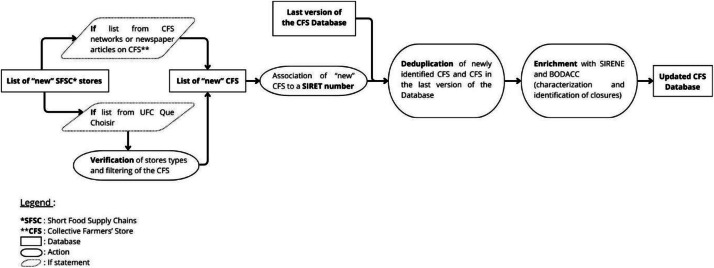
Data validation and enrichment workflow.

This flowchart (Fig. 7) details the process of integrating newly identified Collective Farmers' Stores (CFS), from initial list generation and verification (networks or press articles) to the final database update via SIRET association, deduplication, and administrative enrichment (SIRENE and BODACC).

### 4.5 Type verification

For each establishment, we were not able to verify its full compliance with the legal definition of a “*magasin de producteurs*” as established by the 2014 law, which states that collective governance must be ensured exclusively by farmers and that at least 70 % of the turnover must come from their own production. However, we did verify the existence of collective governance (i.e., at least two producers involved) by consulting newspaper articles, CFS websites, and, in some cases, by contacting the stores directly via email or phone. These sources sometimes provided additional information, such as the opening year of the establishments, or even led to the identification of new collective farmers’ stores. We took advantage of these opportunities and systematically recorded this data.

### 4.6 Identification of SIRET numbers

To connect our database to the national business registry SIRENE, we needed to link each CFS to its corresponding SIRET number, a 14-digit identifier assigned by the INSEE, the French National Institute of Statistics and Economic Studies. To this end, we developed a Python script to query the SIRENE database API, searching for establishments by their name and postal code across various fields (common name, legal name, commercial sign). The SIRENE API was sometimes unable to identify the SIRET of stores, mainly because the common name we knew them as was too far from the one registered in their database. In these cases, we performed manual searches on the stores’ websites as well as on specialized business information platforms (verif.fr, societes.com, infogreffe.fr) to find the missing data.

### 4.7 Enrichment with SIRENE data

Using the identified SIRET numbers, we queried the SIRENE API [14] to extract complementary administrative data: opening date, closure date, address, geographical coordinates, legal status, NAF code (French classification for the Main Activity of a business), and their employee bracket.

### 4.8 Improvement of closure dates via BODACC

To improve the reliability of the dataset, particularly regarding the closure years, we queried the BODACC API (Official Bulletin of Civil and Commercial Notices) [18], which records official business filings. Since this database is updated more frequently than the SIRENE database, its real-time data on judicial liquidations and deregistrations enabled us to refine the closure dates of the stores.

### 4.9 Deduplication

Deduplications of the dataset were performed using a combination of the collective farmers' store names, their municipalities, and their SIRET numbers. Within the framework of our partnership with UFC Que Choisir, they implemented their own internal deduplication process. However, it was still necessary for us to apply an additional deduplication process to ensure the dataset's accuracy.

Our multi-source methodology allowed us to assemble a comprehensive database of 648 collective farmers' stores, including 543 still active in 2024 and 105 that closed over the past fifteen years, offering an up-to-date and thorough overview of this retail model in France.

## Limitations

Although data was gathered from numerous sources, and through various methods, this dataset may not include all collective farmers' stores currently active or that have operated in France. We were particularly successful in identifying establishments that are or have been part of collective farmers' store networks, those featured in local daily newspapers, or those known to UFC Que Choisir members and contributors to the crowdsourcing map published on their website. However, it is possible that stores that have been closed for several years, those with limited public visibility, smaller establishments, or those located in less frequented areas may be absent from this list.

As previously mentioned, the term *“magasin de producteurs”* is legally defined in France. However, despite reviewing a significant amount of information on the stores’ websites and in newspapers, we cannot be entirely certain that each establishment presenting itself as a collective farmers' store fully meets all the criteria outlined in the 2014 law.

## Ethics Statement

The authors have read and followed the ethical requirements for publication in Data in Brief and confirm that the current work does not involve human subjects, animal experiments, or any data collected from social media platforms.

## Credit Author Statement

**Grégori Akermann:** Conceptualization, Methodology, Software, Investigation, Writing - Original Draft, Supervision, Project administration, Funding acquisition; **Manon Pradère:** Methodology, Software, Investigation, Data Curation, Writing - Original Draft, Visualization.

## Declaration of Generative AI and AI-assisted Technologies in the Writing Process

Statement: During the preparation of this work the authors used Claude.ia in order to translate parts of the article from French to English. After using this tool/service, the authors reviewed and edited the content as needed and take full responsibility for the content of the published article.

## Declaration of Competing Interest

The authors declare that they have no known competing financial interests or personal relationships that could have appeared to influence the work reported in this paper.

## Figures and Tables

**Table A.1:** Detailed description of the 22 variables included in the dataset.

variable_name	variable_description	sources_and_methods
idCFS	unique identifier for each establishment using a regional coding system	
commonName	the commonly used name of the establishment	crowdsourcing and newspaper articles
siretNumber	official French business identification number	SIRENE
yearOpening	year when the establishment began operations	aggregated from multiple sources: priority given to opening dates reported in press articles, followed by the ones collected through the BODACC, and finally the ones registered in the SIRENE database
yearClosure	year when the establishment ceased operations, if applicable	aggregated from multiple sources, following the same priority order as year_opening
status	current operational status, either ``active'' or ``inactive''	created from the year_closing column
address	street address of the establishment	crowdsourcing and newspaper articles
postalCode	postal code	crowdsourcing and newspaper articles
municipalityCode	official municipality code	adresse.data.gouv API
municipality	municipality name	crowdsourcing and newspaper articles
frenchDepartment Code	department code	adresse.data.gouv API
frenchDepartment	department name	adresse.data.gouv API
frenchRegion	region name	adresse.data.gouv API
longitude	geographic longitude coordinates	SIRENE and adresse.data.gouv API
latitude	geographic latitude coordinates	SIRENE and adresse.data.gouv API
locationPrecision	indicates the level of accuracy provided for the longitude and latitude data, such as “Street Number” or “Municipality”	created from the address column
legalStatusCode Establishment	numeric code for the legal status of the establishment	SIRENE
legalStatus Establishment	description of the last legal status of the establishment	SIRENE
nafCode Establishment	french classification code for the main economic activity of the establishment	SIRENE
nafLib Establishment	description of the main economic activity of the establishment	SIRENE
employeeBracket CodeEstablishment 2022	employee bracket code as of 2022	SIRENE
employeeBracket Establishment2022	description of employee count bracket	SIRENE

## Data Availability

recherche.data.gouv.frDataset French Collective Farmers' Stores (Original data).

## Footnotes

1 UFC Que Choisir has specific criteria that a store must meet to appear on the crowdsourcing map, such as offering a diverse range of products, a requirement that not all collective farmers' stores in our dataset fulfill.

## References

[1] M. Pradère, G. Akermann, I. Bourcier, G. Caret, M. Perier-Dulhoste, L. Hamon, Dataset French Collective Farmers' Stores", Recherche Data Gouv, 10.57745/YX1JGK

[2] Renting H., Marsden T.K., Banks J. (2003). Understanding alternative food networks: exploring the role of short food supply chains in rural development. Environ. Plan. A.

[3] Chiffoleau Y., Dourian T. (2020). Sustainable food supply chains: is shortening the answer? A literature review for a research and innovation agenda. Sustainability.

[4] Chiffoleau Y., Prévost B. (2012). Les circuits courts, des innovations sociales pour une alimentation durable dans les territoires. Norois.

[5] Chiffoleau Y. (2019).

[6] Jaouen A., Jaeck M., Joly C., Kessari M. (2020). Les magasins de producteurs: vers un renouveau de l’action collective des PME agricoles. Rev. Int. P.M.E.

[7] French Law n° 2014-344 of March 17, 2014 relating to consumption. https://www.legifrance.gouv.fr/loda/id/JORFTEXT000028738036 (accessed 30 March 2025).

[8] Kessari M., Joly C., Jaouen A., Jaeck M. (2020). Alternative food networks: good practices for sustainable performance. J. Mark. Manag..

[9] Vonthron S., Devillet G. (2023).

[10] Vonthron S., Perrin C., Soulard C.T. (2023). Mapping the food environment: The reliability of volunteered geographical information and institutional data sources in France. Trans. GIS.

[11] Creurer Q., Vonthron S., Hilal M., Charreire H., Napoleone C., Pradere M., Sanz-Sanz E. (2025). Decoding France's food environment combining data bases to characterize the food environment in France. Data Brief.

[12] Observatory of Territorialized Food Systems. http://www.obsat.org (accessed 30 March 2025).

[13] (30 March 2025). RMT Local Food. https://www.rmt-alimentation-locale.org/(accessed.

[14] Sirene. https://www.sirene.fr/(accessed 30 March 2025).

[15] Free map of short food supply chains. https://www.quechoisir.org/carte-interactive-circuit-court-n97688/ (accessed 30 March 2025).

[16] AFIPaR. https://www.afipar.org/(accessed 30 March 2025).

[17] Réseau des boutiques paysannes. https://boutiquespaysannes.fr/(accessed 30 March 2025)

[18] Official Bulletin of Civil and Commercial Announcements (BODACC). https://www.bodacc.fr/(accessed 30 March 2025).

